# Individualized, perioperative, hemodynamic goal-directed therapy in major abdominal surgery (iPEGASUS trial): study protocol for a randomized controlled trial

**DOI:** 10.1186/s13063-018-2620-9

**Published:** 2018-05-09

**Authors:** Sandra Funcke, Bernd Saugel, Christian Koch, Dagmar Schulte, Thomas Zajonz, Michael Sander, Angelo Gratarola, Lorenzo Ball, Paolo Pelosi, Savino Spadaro, Riccardo Ragazzi, Carlo Alberto Volta, Thomas Mencke, Amelie Zitzmann, Benedikt Neukirch, Gonzalo Azparren, Marta Giné, Vicky Moral, Hans Otto Pinnschmidt, Oscar Díaz-Cambronero, Maria Jose Alberola Estelles, Marisol Echeverri Velez, Maria Vila Montañes, Javier Belda, Marina Soro, Jaume Puig, Daniel Arnulf Reuter, Sebastian Alois Haas

**Affiliations:** 1 Department of Anesthesiology, Center of Anesthesiology and Intensive Care Medicine, University Medical Center Hamburg-Eppendorf, 20246 Hamburg, Germany; 2 Department of Anesthesiology, Intensive Care Medicine and Pain Therapy, Universitätsklinikum Giessen und Marburg GmbH, 35392 Giessen, Germany; 3 Department of Surgical Sciences and Integrated Diagnostics, San Martino Policlinico Hospital, IRCCS for Oncology, University of Genoa, Genoa, Italy; 4 Department of Anesthesia and Intensive Care, University of Ferrara, Sant Anna Hospital, Via Aldo Moro, 8, 44121 Ferrara, Italy; 5 Department of Anesthesia and Intensive Care Medicine, University of Rostock, Schillingallee 35, 18057 Rostock, Germany; 6 Department of Anesthesiology, Hospital Santa Creu i Sant Pau, C/ Mas Casanovas 90, 08041 Barcelona, Spain; 7 Institute of Medical Biometry and Epidemiology, University Medical Center Hamburg-Eppendorf, 20246 Hamburg, Germany; 8 Department of Anaesthesiology, Perioperative Medicine Research Group, Hospital Universitari i Politecnic La Fe, Instituto de Investigación Sanitaria La Fe (IIS laFe), Valencia, Spain; 9 Department of Anesthesiology, Hospital Clínico Universitario de Valencia, Avda. Blasco Ibañez 17, 46010 Valencia, Spain

**Keywords:** Postoperative morbidity, Mortality, Hemodynamic optimization, Individualized medicine, Quality of life

## Abstract

**Background:**

Postoperative morbidity and mortality in patients undergoing surgery is high, especially in patients who are at risk of complications and undergoing major surgery. We hypothesize that perioperative, algorithm-driven, hemodynamic therapy based on individualized fluid status and cardiac output optimization is able to reduce mortality and postoperative moderate and severe complications as a major determinant of the patients’ postoperative quality of life, as well as health care costs.

**Methods/design:**

This is a multi-center, international, prospective, randomized trial in 380 patients undergoing major abdominal surgery including visceral, urological, and gynecological operations. Eligible patients will be randomly allocated to two treatment arms within the participating centers. Patients of the intervention group will be treated perioperatively following a specific hemodynamic therapy algorithm based on pulse-pressure variation (PPV) and individualized optimization of cardiac output assessed by pulse-contour analysis (ProAQT© device; Pulsion Medical Systems, Feldkirchen, Germany). Patients in the control group will be treated according to standard local care based on established basic hemodynamic treatment. The primary endpoint is a composite comprising the occurrence of moderate or severe postoperative complications or death within 28 days post surgery. Secondary endpoints are: (1) the number of moderate and severe postoperative complications in total, per patient and for each individual complication; (2) the occurrence of at least one of these complications on days 1, 3, 5, 7, and 28 in total and for every complication; (3) the days alive and free of mechanical ventilation, vasopressor therapy and renal replacement therapy, length of intensive care unit, and hospital stay at day 7 and day 28; and (4) mortality and quality of life, assessed by the EQ-5D-5L™ questionnaire, after 6 months.

**Discussion:**

This is a large, international randomized controlled study evaluating the effect of perioperative, individualized, algorithm-driven ,hemodynamic optimization on postoperative morbidity and mortality.

**Trial registration:**

Trial registration: NCT03021525. Registered on 12 January 2017.

**Electronic supplementary material:**

The online version of this article (10.1186/s13063-018-2620-9) contains supplementary material, which is available to authorized users.

## Background

In high-risk surgical patients, rates of postoperative complications range from 25% up to more than 40% [1, 2]. These patients not only have reduced functional independence, quality of life and long-term survival [3, 4], moreover, the treatment of postsurgical complications is extremely expensive. In the US the average extra cost for treating a patient developing one or more complication is approximately US$18,000 [1, 5, 6]. Therefore, reduction of postsurgical complications with fewer days in an intensive care unit, fewer days on organ support, reduction of resource utilization and reduction of hospital stay would reduce costs also for the health care system. Inadequate supply of oxygen is one of the major factors leading to postoperative organ dysfunction and complications. Oxygen supply is mainly determined by cardiac output rather than blood pressure, which, however, has been the primary hemodynamic target during surgery for decades and still is. In this context hemodynamic goal-directed therapy, focusing on an optimization of fluid status and cardiac output, is believed to have the ability to reduce postoperative complications. Since methods like thermodilution, arterial pulse-contour analysis or esophageal Doppler allow routine measurement of cardiac output, several single-center studies have brought up the first evidence that perioperative, algorithm-driven optimization of cardiac output can reduce postoperative complications and improve outcome [7–11]. Also meta-analyses have demonstrated a reduction of morbidity in patients undergoing high-risk surgery [12–17]. However, comparability of those studies is limited due to inhomogeneity in population and therapeutic approaches. Moreover, in most published trials so far, hemodynamic goals had been predefined independently from the individual need of the single patient and further generalized to an entire study population, i.e., either dedicated values of stroke volume or cardiac output, or a standardized maximization of stroke volume by volume loading were used for all patients.

The focus in previous studies was put on implementation of fixed values of hemodynamic parameters, disregarding the single patient’s individual cardiovascular capacities, especially the individual range of cardiac output.

The hypothesis of the planned clinical trial is that perioperative hemodynamic optimization based on the novel approach of individualization of hemodynamic therapy can reduce postoperative morbidity and mortality.

## Methods/design

### Justification of the study

The present study aims to evaluate the impact of perioperative, algorithm-driven, hemodynamic therapy based on individualized fluid status and individualized cardiac output optimization on postoperative moderate and severe complications and mortality. The study is justified by the high rate of postoperative complications as a major determinant for the reduction of quality of life for the affected patients and high costs for the health system which may be reduced by individualized, perioperative hemodynamic therapy.

### Study design

This is a prospective, multi-center, international, randomized controlled clinical trial in 380 patients scheduled for major abdominal surgery in six European centers having a large number of patients eligible for study inclusion (see Appendix). The study coordination will be conducted by a team of principle investigators (PI), Daniel A. Reuter, Sebastian A. Haas, Sandra Funcke, and Bernd Saugel, from the University Medical Center Hamburg-Eppendorf, Germany and from the Rostock University Medical Center, Germany.

**Fig. 1:**
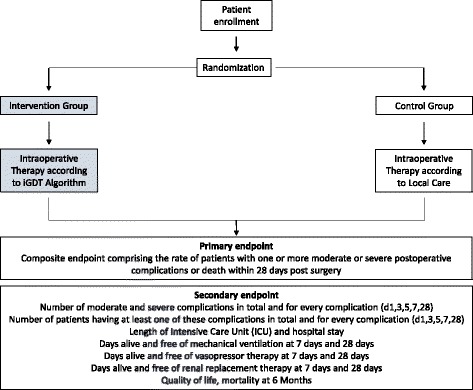
Study flow chart. iGDT: individualized, goal-directed therapy

The trial was designed in accordance with the fundamental principles established in the Declaration of Helsinki and within the requirements established by the German legislation in the field of biomedical research, the protection of personal data, and bioethics. The study was registered in January 2017 (received on 12 January 2017) at https://clinicaltrials.gov with the identification number NCT03021525. The study was approved by the Ethics Committee Giessen, Germany in February 2017 acting as the overall Ethical Review Board for this study (Additional file 1). Further, we can specify that all participating centers will obtain the approval from the local Ethical Review Board according to individual legislation regulations. Before inclusion into the study, written informed consent from the patient will be obtained (Additional file 2). See Additional file 3 for the Standard Protocol Items: Recommendations for Interventional Trials (SPIRIT) Checklist of the study protocol and Fig 1. for the adapted SPIRIT Figure.

After an initial meeting of the principle investigators (PIs) together with all local main investigators (LMI) the centers are visited at the partner hospital and trained with regard to the process of randomization, enrollment, data acquisition, and treatment strategies.

### Study population

To be eligible for inclusion into this study (day 0), each patient must fulfill all the inclusion criteria during screening and prior to enrollment into the trial.

#### Inclusion criteria

To ensure the presence of marked surgical trauma, open visceral, urological, and gynecological surgery are covered by this study. Expected duration of surgery must be ≥ 120 min and the requirement of volume therapy must be expected to be ≥ 2 l. Risk for any postoperative complications must be ≥ 10% as assessed preoperatively by the ACS-NSQIP (American College of Surgery – National Surgical Quality Improvement Program, www.acsnsqip.org) risk calculator [4, 18, 19].

#### Exclusion criteria

Patients aged < 18 years, use of a laparoscopic approach, patients who are not in sinus rhythm, patients having highly impaired left ventricular function (ejection fraction < 30%) or severe aortic valve stenosis (aortic valve area < 1 cm^2^, mean gradient > 40 mmHg), pregnant women, emergency surgeries (surgery required within 24 h), primarily vascular surgery, patients suffering from septic shock, patients having pheochromocytoma, patients suffering from non-cardiac chest pain, patients suffering from anuric renal failure, those refusing to consent, patients receiving palliative treatment only (likely to die within 6 months) and patients suffering from acute myocardial ischemia (within 30 days before randomization) are excluded from the study. Further, in case that clinicians intended to use cardiac output monitoring for clinical reasons patients should also not be included in the study.

#### Randomization

The use of an electronic Case Report Form (eCRF) including randomization software ensures a safe and prompt randomization via computer. Participants will be centrally allocated to treatment groups (allocation ratio: 1:1) by the eCRF-generated randomization procedure (randomization in blocks of 6). This ensures a balanced allocation with respect to centers and treatment arms. The study is summarized in Fig. 1.

## General care and procedures for the control and intervention groups

### General information on patients’ treatment

Treatment besides hemodynamic optimization in both the control and the intervention group is defined based on the respective guidelines and recommendations of the medical societies to avoid extremes of clinical practice. Oxygenation will be targeted for a SpO_2_ of 94% or higher by choosing an appropriate level of inspired oxygen. The tidal volume will be set at 6–8 ml/kg and PEEP at 0–10 cm H_2_O according to clinical needs and the decision of the physician in charge. The target for heart rate is below 100 bpm, the target range for mean arterial blood pressure is 65 mmHg or higher, using vasopressors as required. Core temperature will be maintained at > 36 C by continuous heat application to the patient and fluid warming. Choice of fluid is determined on the basis of the recent guidelines [20] for intravascular volume therapy. Arterial or venous blood samples are taken regularly for assessment of hemoglobin, oxygenation, decarboxylation, electrolytes, and lactate. An epidural catheter is placed before induction of anesthesia at the discretion of the treating anesthesiologist. Perioperative analgesia will be provided by epidural or intravenous infusion of analgesics according to the standards of the participating centers. Blood products will be transfused according to the Guidelines from the European Society of Anesthesiology (for an overview see Additional file 4: Table S1) [21].

### Treatment of the control group

Patients in the control group will be treated according to established basic treatment goals as described in the general information on patient treatment (heart rate < 100 bpm, mean arterial pressure > 65 mmHg, SpO_2_ > 94%, and core temperature > 36 C). In the control group, no specific vasopressor is prescribed to achieve the mean arterial pressure goal and the choice of drug is at the discretion of the treating anesthesiologist. Basic anesthesiological monitoring by five-lead electrocardiogram, pulse oximetry, non-invasive blood pressure monitoring and capnography is performed in every patient. Placement of an arterial and central venous line is at the discretion of the treating clinician, as is the decision to administer fluids and catecholamines. After extubation, hemodynamic treatment in the recovery room is at the discretion of the treating anesthesiologist either by administration of fluids, catecholamines, or other drugs as required.

### Treatment of the intervention group

The trial intervention period will commence from the induction of anesthesia until 8 h after surgery, or until discharge from the postoperative intensive or intermediate care unit. Drugs of choice are dobutamine for inotropic support and norepinephrine as a vasopressor. No other catecholamines should be used in the intervention group. Hemodynamic management is performed according to standard treatment until an arterial catheter is placed. The signal from the arterial catheter is then processed by non-calibrated pulse-contour analysis (ProAQT©; Pulsion Medical Systems, Feldkirchen, Germany) for pulse-pressure variation (PPV) and cardica index (CI) measurement. A fluid challenge will be performed by infusion of 500 ml in < 15 min. The choice of fluid in case of indicated fluid loading is determined on the basis of the recent guidelines for volume therapy [20].

#### Initial assessment of individually optimized CI

**Fig. 2:**
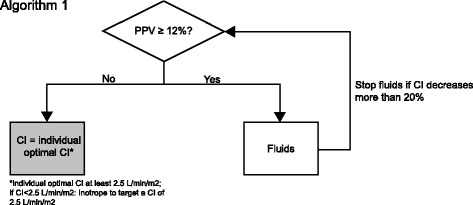
Algorithm 1. Intervention algorithm of initial assessment of individually optimized Cardiac Index (CI). CI: Cardiac index; PPV: Pulse pressure variation

When arterial pulse-contour analysis is implemented, patients receive an initial hemodynamic assessment based on PPV and CI in order to identify individual values of optimal CI as shown in algorithm 1 (Fig. 2).

First, fluid status is optimized by fluid loading (500 ml in < 15 min) until PPV is < 12%. At least 15 min after induction of general anesthesia, after individual optimization of the fluid status, the patient’s individual optimal CI in the specific situation of general anesthesia is defined and used as the individual hemodynamic goal until the end of the intervention period. Only if this value is below 2.5 l/min/m^2^ inotropes are administered to reach this minimum CI to prevent patients from developing a low cardiac output.

#### Hemodynamic assessment during mechanical ventilation

**Fig. 3:**
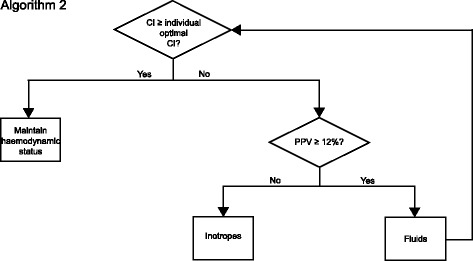
Algorithm 2. Intervention algorithm during mechanical ventilation. CI: Cardiac index; PPV: Pulse pressure variation

After the initial determination of the individual optimal CI, further hemodynamic assessment during mechanical ventilation and surgery is performed every 15 min, or if mechanical ventilation is continued after surgery every 30 min, or if required due to hemodynamic instability at any time according to algorithm 2 (Fig. 3). Hemodynamic parameters are documented every 30 min during surgery and every 60 min postoperatively.

If the CI is below the initially assessed individual optimal CI, again fluid status is evaluated based on PPV. Fluids are administered as long as PPV is higher than 12% and the CI increases. Again, inotropes are used when fluid optimization does not result in realization of the individual optimal CI. This treatment algorithm is always reassessed when the CI drops below the individually optimized CI.

#### Hemodynamic assessment after extubation (maximum 8 h after surgery)

**Fig. 4:**
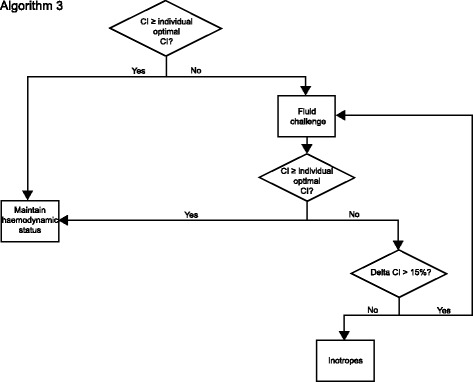
Algorithm 3. Intervention algorithm during spontaneous breathing. CI: Cardiac index; PPV: Pulse pressure variation

Because of spontaneous breathing, after extubation PPV is not usable anymore for fluid status evaluation. Hemodynamic assessment is then performed according to algorithm 3 (Fig. 4). Hemodynamic assessment after extubation is repeated every 30 min, or if required due to hemodynamic instability at any time according to algorithm 3. Hemodynamic parameters are documented every 60 min.

When the CI is below the individually determined optimal CI, a fluid challenge (500 ml in < 15 min) is performed. If the increase of CI is higher than 15%, a fluid challenge of 500 ml is repeated. If the increase of the CI is below 15%, or the CI even decreases, inotropes should be started or increased.

### Primary and secondary outcomes

#### Primary endpoint

Considering the high relevance for the individual patient and for society, the postoperative outcome is most appropriately reflected by a combination of morbidity and mortality. Therefore, the primary endpoint is a composite comprising the rate of patients with one or more moderate or severe postoperative complications or death within 28 days post surgery. Moderate and severe postoperative complications are defined by 22 single-organ outcome failures based on a consensus statement of the European Society of Anesthesiology (ESA) – European Society of Intensive Care Medicine (ESICM) joint taskforce on perioperative outcome measures [22]. A summary of the included organ failures and the severity grading is given in Additional file 5: Table S2.

#### Secondary endpoints

Secondary endpoints are: (1) the number of moderate and severe postoperative complications in total, per patient and for each individual complication; (2) the occurrence of at least one of these complications on days 1, 3, 5, 7, and 28 in total and for every complication. The additional secondary endpoints include (3) days alive and free of mechanical ventilation, vasopressor therapy and renal replacement therapy, length of ICU and hospital stay at day 7 and day 28 further characterize perioperative morbidity and its socioeconomic impacts. (4) For the assessment of quality of life, an interview (personal or by telephone) based on the EQ-5D-5L™ questionnaire [23] is performed at the time point of enrollment and 6 months after surgery. Mortality is assessed 6 months after surgery. All the secondary endpoints mentioned above will be recorded at the defined time points, but will be analyzed at the end of the study.

### Intervention scheme

**Fig. 5:**
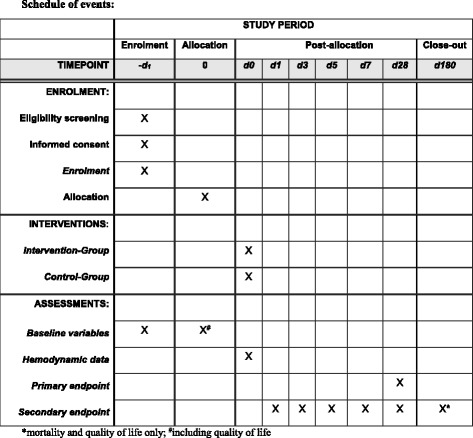
Schedule of events

The intervention schemes are given in Figs. 2, 3, and 4. The schedule of events is summarized in Fig. 5.

### Documentation and data acquisition

Data will be obtained by a web-based eCRF. Study centers will be trained on the use of the eCRF before initiating a patient’s enrollment. To enter a patient into the iPEGASUS trial, research staff at the site will log on to a secure, web-based randomization system via a link to the respective website and complete the patient’s details to obtain a unique patient number and allocation to a treatment group. The local main investigator (LMI) will then be informed about the patient’s enrollment. For correct patient data follow-up only the LMI will set up a list with the possibility to retrace the patient’s history. Personalized data will be handled by the LMI according to Good Clinical Practice (GCP) requirements. In contrast, data in the eCRF, visible only for the coordinating investigators, will be completely anonymized and not be re-traceable. Data management of the eCRF will be performed by a company specialized in full service study data management (MedSurv GmbH, Nidderau, Germany).

To reduce bias resulting from lack of experience in the use of the protocol, the first two enrolled patients of each center (one allocated to the control group, one allocated to the intervention group) will be withdrawn from the study. Source data verification, quality assurance and monitoring of data during on-site monitoring visits will be performed by the dedicated monitoring team with appropriate frequency according to center performance. The monitoring team will be provided by the Hamburg-Eppendorf University Medical Center.

#### Methods against bias

Due to the nature of the intervention, blinding to all types of staff involved in the study is not possible. Primary and secondary outcome parameters will be assessed by a local investigator, on days 1, 3, 5, 7, and on day 28, blinded to allocation. Moderate and severe complications will additionally be verified by the LMI or a designee. Source data verification will be performed by the monitors by regular on-site monitoring visits. To minimize bias provoked by learning principles of goal-directed therapy in the past, and thereby influencing control group treatment, predominately hospitals without prior regular use of cardiac output monitoring in the respective procedures were selected. Further, the inclusion of non-university centers will allow better comparison of the study group to the broad clinical reality.

#### Compliance/rate of loss to follow-up

After enrollment of the patient’s address, telephone number, and contact information of the primary care physician, a relative of the patient will also be registered enabling us to maintain contact. If patients do not reach the goals of the inclusion criteria (time of surgery > 120 min or volume therapy > 2 l) they will be excluded from the study subsequently. Visits by a local investigator will be performed on days 1, 3, 5, 7, and 28 to assess postoperative complications necessary for primary and secondary endpoints. However, if patients are discharged from hospital to home, to a secondary hospital, or to a rehabilitation facility before day 28, final determination of all parameters relevant for the primary and the secondary endpoints will be assessed on day 28 by a structured telephone interview with the secondary hospital, the patient or the rehabilitation facility or the primary care physician. Due to the lack of subsequent examinations of the patient after day 28, loss to follow-up is unlikely. However, to reflect an unscheduled loss to follow-up an overall dropout rate of 10% is already included in the sample size calculation.

### Sample size calculation and statistical analysis

#### Sample size

According to previous published data [24] assessing the impact of hemodynamic goal-directed therapy, we assume that the primary outcome will occur in 48% of the control group and in 33% of the intervention group. The null hypothesis of the study is that individualized, goal-directed therapy does not lead to a statistically significant difference in morbidity and mortality at day 28 after major abdominal surgery. Assuming in this null hypothesis (H0) that the two groups are not significantly different from each other at the significance level α = 0.05 with respect to the primary outcome, 334 patients are required to have an 80% chance of detecting a difference between the control group and the experimental group of 15%. Therefore, 167 patients per group would have to be enrolled into the study. Taking into account a dropout rate of about 10% and exclusion of the first two patients in every center, a number of *n* = 380 patients is necessary.

#### Primary and secondary endpoints

Baseline sample characteristics will be shown for all randomized patients treatment-wise and for pooled data. For categorical variables, category frequency counts and percentages will be shown. For continuous variables, the arithmetic mean, median, 1st and 3rd quartiles, minimum and maximum values will be presented. Follow-up data will be given in line plots allowing visual comparisons between treatment groups.

#### Primary analysis

For the primary endpoint “rate of moderate or severe postoperative complication or death within 28 days post surgery,” the fixed effect of treatment and the random effect of clinic and the treatment slope will be estimated by a binary logistic regression model. The resulting odds ratio with corresponding 95% confidence intervals (CI) and *p* values will be tabulated. Estimated marginal frequencies and their 95% CIs will be presented graphically. Superiority of the individualized hemodynamic therapy is considered established if the upper margin of the 95% CI of the corresponding odds ratio lies below 1.

#### Secondary analyses

The composite endpoint “occurrence of moderate or severe postoperative complication or death” and the secondary endpoints “postoperative morbidity,” “occurrence of moderate or severe complications,” “occurrence of individual complications” and “number of complications per patient” will be analyzed via longitudinal binary logistic regression models, treating clinic, treatment slope and patient within clinic as random effects. Treatment, frequencies over time (FU) and their interaction will be considered fixed effects. Odds ratios, their 95% CIs and *p* values will be tabulated. Estimated marginal FU and their 95% CIs will be presented graphically.

## Discussion

Reduction of perioperative mortality and morbidity is greatly required due to the massive benefit for the patients and the health care system. In Europe, overall mortality after surgery has been reported to be up to 4% [2] and after high-risk surgery or in high-risk patients it has to be assumed to be even higher. Morbidity or postoperative complications, which are potentially triggered by inadequate oxygen supply to the peripheral organs, have to be seen as the primary cause for reduction of quality of life and the immense health care costs ensuing. Every percentage point reduction of postoperative complications could save approximately 180 million to US$270 million [5]. In a recent multi-center trial the achievement of adequate preoperative oxygen delivery was associated with a lower postoperative morbidity compared to the standard of care [25]. In this context a various number of meta-analyses evaluating the impact of hemodynamic goal-directed therapy, and thereby optimizing oxygen supply, have already demonstrated reduction of morbidity in patients undergoing high-risk surgery [12–17]. However, meta-analyses are frequently limited in their statement due to inhomogeneity in population and therapeutic approaches. In published trials, stroke volume or cardiac output have been predefined, standardized and generalized to a total cohort of patients not taking into account the single patient’s individual cardiovascular needs and capacities. In the largest randomized multi-center study on goal-directed therapy published so far, the focus of the intervention protocol was maximization of stroke volume by repetitive fluid administration without using functional parameters of preload supplemented by a fixed dose of inotropes. In this trial the focus was put on maximizing stroke volume. However, this large multi-center trial did not significantly reduce the composite primary endpoint of morbidity and mortality [26]. In contrast to this approach, in the iPEGASUS trial, the protocol is not targeting a maximized stroke volume. In the iPEGASUS trial, individual CI assessment is primarily based on PPV-guided volume status optimization followed by defining the achieved CI as “individually optimized CI” under optimized volume status conditions. This “individually optimized CI” is used as the CI target during the total perioperative period and volume and inotrope application is only performed when this “individually optimized CI” is not reached.

The hypothesis of the planned clinical trial is that perioperative hemodynamic optimization, based on the novel approach of individualization of hemodynamic therapy, can reduce postoperative morbidity and also mortality. A previous study focusing on a PPV-guided CI individualization and, in contrast to iPEGASUS, using PPV-based volume loading, even when the individualized CI was reached, demonstrated a reduction in postoperative complications [24]. However, this finding is now evaluated in a larger randomized controlled, multi-center study using a modified intervention protocol. Thus, in the proposed study, hemodynamic therapy is tailored individually to each patient, based on optimization of fluid status by the functional parameter PPV and based on this individually titrated goal of CI. Individualization of therapy is a key factor for successful treatment, especially when reduction of morbidity and mortality is targeted. Therefore, the iPEGASUS trial further develops the concept of hemodynamic goal-directed therapy to individually set goals and is designed to assess its impact on mortality and morbidity.

## Trial status

Recruitment started in August 2017 and is anticipated to be completed by the end of 2018.

### Additional files

Additional file 1: Ethical approval. (PDF 386 kb)

Additional file 2: Written informed consent. (PDF 53 kb)

Additional file 3: SPIRIT 2013 Checklist. (DOC 121 kb)

Additional file 4: **Table S1.** Management of severe bleeding – Extract from the *Guidelines from the European Society of Anaesthesiology*. (PDF 258 kb)

Additional file 5: **Table S2.** Overview of Single Organ Outcome Failures and Severity Grading. (PDF 182 kb)

## Appendix

These are the collaborators in the European centers initiated for patient recruitment and enrollment:

1. Policlinico San Martino Hospital, IRCCS for Oncology, University of Genoa, Genoa, Italy
2. Department of Morphology, Surgery and Experimental Medicine, Intensive Care Unit University of Ferrara, Sant’Anna Hospital, Via Aldo Moro, 8, 44,121, Ferrara, Italy
3. Department of Anesthesiology, Intensive Care Medicine and Pain Therapy, Universitätsklinikum Giessen und Marburg GmbH, Giessen, Germany
4. Department of Anesthesia and Intensive Care Medicine, University of Rostock, Schillingallee 35, Rostock 18,057, Germany
5. Department of Anesthesiology, Hospital Universitari i Politécnic La Fe, Valencia, Spain
6. Department of Anesthesiology, Hospital Santa Creu i Sant Pau, C/ Mas Casanovas 90, 08041, Barcelona, Spain

## Abbreviations

CI: Cardiac Index
eCRF: Electronic Case Report Form
ERB: Ethical Review Board
ESA: European Society of Anesthesiology
ESICM: European Society of Intensive Care Medicine
ICU: Intensive care unit
iGDT: Individualized, goal-directed therapy
LMI: Local main investigator
MAP: Mean arterial pressure
PI: principle investigators
PPV: Pulse-pressure variation
