# Supplementary material for: Individualized, perioperative, hemodynamic goal-directed therapy in major abdominal surgery (iPEGASUS trial): study protocol for a randomized controlled trial
Source: Trials. 2018 May 9;19:273. doi: 10.1186/s13063-018-2620-9 (PMC5944092; doi:10.1186/s13063-018-2620-9)
Supplement: Supplementary file 2 — Written informed consent. (PDF 53 kb) [file 13063_2018_2620_MOESM2_ESM.pdf]

Prüfstelle: Klinik für Anästhesiologie, Operative Intensivmedizin und Schmerztherapie der Justus-Liebig Universität Gießen
Prüfarzt: Univ. Prof. Dr. med. Michael Sander

„iPEGASUS – Individualisierte, zielgerichtete Herzkreislauf-Therapie bei großen bauchchirurgischen Operationen – Eine prospektive, randomisierte, klinische Studie“

Sehr geehrte Patientin, sehr geehrter Patient,

wir möchten Sie fragen, ob Sie bereit sind, an der nachfolgend beschriebenen klinischen Studie teilzunehmen.

Klinische Studien sind notwendig, um Erkenntnisse über die Wirksamkeit und Verträglichkeit von medizinischen Behandlungsmethoden zu gewinnen oder zu erweitern. Die klinische Studie, die wir Ihnen hier vorstellen, wurde von der zuständigen Ethikkommission zustimmend bewertet. Diese klinische Prüfung wird an der Universität Gießen und an 5 weiteren europäischen Zentren durchgeführt; es sollen insgesamt ungefähr 380 Personen daran teilnehmen. Die Studie wird veranlasst, organisiert durch die Universitätsklinikum Gießen und durch das Universitätsklinikum Hamburg-Eppendorf und finanziert durch PULSION Medical Systems SE, Hans-Riedl Str. 2, 85622 Feldkirchen, dem Verantwortlichen für diese Studie.

Ihre Teilnahme an dieser klinischen Studie ist freiwillig. Sie werden in diese Prüfung also nur dann einbezogen, wenn Sie dazu schriftlich Ihre Einwilligung erklären. Sofern Sie nicht an der klinischen Studie teilnehmen oder später aus ihr ausscheiden möchten, erwachsen Ihnen daraus keine Nachteile.

Sie wurden bereits auf die geplante Studie angesprochen. Der nachfolgende Text soll Ihnen die Ziele und den Ablauf erläutern. Anschließend wird ein Prüfarzt das Aufklärungsgespräch mit Ihnen führen. Bitte zögern Sie nicht, alle Punkte anzusprechen, die Ihnen unklar sind. Sie werden danach ausreichend Bedenkzeit erhalten, um über Ihre Teilnahme zu entscheiden.

1. Warum wird diese Studie durchgeführt?

Im Rahmen eines größeren bauchchirurgischen Eingriffs ist mit Flüssigkeitsverlusten aus dem Körper und –verschiebungen innerhalb des Körpers zu rechnen. Diese werden regelhaft durch Flüssigkeitsgabe in das Gefäßsystem ausgeglichen. Dadurch wird sichergestellt, dass die Versorgung der Organe und Gewebe mit Sauerstoff und Nährstoffen gewährleistet ist. Eine Unterversorgung könnte zu Komplikationen von Organfunktionen und längerfristigen Einschränkungen der Lebensqualität führen. Ziel ist es daher, durch exakt gesteuerte Flüssigkeitsgabe eine optimierte Pumpleistung des Herzens zu erreichen und damit eine bestmögliche Versorgung aller Organsysteme, gleichzeitig aber auch eine übermäßige Flüssigkeitsgabe mit der Gefahr von Wasseransammlungen im Körper zu verhindern. Diese Einschätzung des Flüssigkeits-Bedarfs während einer Operation wird traditionell durch klinische Parameter (Blutdruck, Herzfrequenz, Flüssigkeitsfüllung der Halsvenen usw.) vorgenommen. In den letzten Jahren sind allerdings technische Geräte entwickelt worden, die anhand der Pulskurve der arteriellen Blutdruckmessung weitere Parameter zur Einschätzung des Flüssigkeitsbedarfs bereitstellen. In der vorlie-

genden Studie soll untersucht werden, ob eine Flüssigkeitsgabe, die anhand dieser Pulskurve orientiert ist, einen Vorteil hinsichtlich des Auftretens von Komplikationen nach größeren Operationen im Bauchraum bietet. Von der Durchführung der vorgesehenen klinischen Prüfung erhoffen wir uns, dass wir zukünftig Konzepte zur optimalen Flüssigkeitsgabe während einer Operation zur Verfügung stellen können, um die Anzahl an Komplikationen nach einer Operation weiter zu minimieren.

2. Werde ich auf jeden Fall mit der neuen Behandlungsmethode behandelt?

Im Rahmen dieser klinischen Prüfung wird das Standard-Flüssigkeits- und Kreislaufmanagement mit einer individualisierten, d.h. genau auf die jeweiligen Bedürfnisse jedes einzelnen Patienten zugeschnittenen, zielgerichteten Flüssigkeitstherapie verglichen. Diese Therapieform hat in der Literatur den Namen „Hemodynamic Goal-Directed Therapy - GDT“ erhalten und wurde bereits bei intensivmedizinischen Krankheitsbildern erfolgreich eingesetzt. Im Falle Ihrer Teilnahme werden Sie entweder mit der Standard-Therape oder mit der individualisierten, zielgerichteten Therapie behandelt. Welche der Behandlungen Sie im Falle Ihrer Teilnahme erhalten, entscheidet der Zufall (dieses Verfahren wird Randomisierung genannt). Die Wahrscheinlichkeit, eine individualisierte, zielgerichtete Flüssigkeitstherapie zu erhalten, beträgt 50%.

Zur optimalen, objektiven Gewinnung von Studiendaten ist es notwendig, dass weder Sie noch Ihr Prüfarzt wissen, welche Behandlung Sie erhalten (dieses Verfahren wird als „doppelblind“ bezeichnet). Dass Ihr Prüfarzt die Art Ihrer Behandlung nicht kennt, ist jedoch bei dieser Studie nicht möglich, da er selber die Art der Behandlung durchführen muss. Die Auswertung der Ergebnisse wird jedoch von einem anderen Prüfarzt durchgeführt, der die Art der Behandlung nicht kennt, damit die Qualität der Studiendaten möglichst hoch gehalten werden kann. Sollte es dennoch aus Sicherheitsgründen notwendig sein, kann unverzüglich festgestellt werden, welche Behandlung Sie erhalten haben.

3. Wie ist der Ablauf der Studie und was muss ich bei Teilnahme beachten?

Bei Aufnahme in diese klinische Studie wird die Vorgeschichte Ihrer Krankheit erhoben und Sie werden einer umfassenden ärztlichen Untersuchung unterzogen. Dazu gehört insbesondere eine allgemeine Risikoabschätzung, wie groß die theoretische Wahrscheinlichkeit ist, eine Organkomplikation nach der Operation zu erleiden. Die Möglichkeit Ihrer weiteren Teilnahme an dieser klinischen Studie wird von den Ergebnissen dieser Voruntersuchung abhängen.

Bei Teilnahme an der Studie sind für Sie zunächst keinerlei Veränderungen bemerkbar. Es werden keine zusätzlichen Interventionen, Blutabnahmen oder ähnliches an Ihnen vorgenommen, die nicht auch bei der Standardtherapie durchgeführt worden wären. Nach Einleitung der Narkose wird lediglich ein weiteres Gerät zu Kreislaufüberwachung in Ihre bestehende Kreisluafüberwachung zwischengeschaltet, welches eine detailliertere Überwachung Ihrer Kreislauffunktion und Ihres Flüssigkeitshaushaltes erlaubt. Anhand dieser zusätzlichen Daten wird auf der Basis eines Therapiealgorithmus eine individuellere und spezifischere Flüssigkeits und Kreislauftherapie durchgeführt. Diese individualisierte und zielgerichtete Therapieform wird spätestens 8 Stunden nach Ihrer Operation beendet. Anschließend erfolgt lediglich noch eine Nachbeobachtung und Dokumentation Ihres Heilungsverlaufes. Gerne würden wir uns nach 6 Monaten einmal telefonisch bei Ihnen melden, um Ihnen in einem ca. 5 minütigem Gespräch wenige Fragen zu Ihrer Lebensqualität zu stellen.

4. Welchen persönlichen Nutzen habe ich von der Teilnahme an der Studie?

Wenn Sie die neue Behandlungsmethode erhalten, kann möglicherweise Ihre Erkrankung positiv beeinflusst werden, so dass die Wahrscheinlichkeit, Komplikationen nach der Operation zu entwickeln, möglicherweise minimiert wird. Da die Wirksamkeit der neuen Behandlungsmethode noch nicht erwiesen ist, ist es jedoch auch möglich, dass Sie durch Ihre Teilnahme an dieser klinischen Prüfung nicht den erhofften Nutzen haben.

Wenn Sie die Standardtherapie erhalten, verändern sich Ihre Behandlungsaussichten durch die Teilnahme an der Studie im Vergleich zur Nichtteilnahme voraussichtlich nicht.

5. Welche Risiken sind mit der Teilnahme an der Studie verbunden?

Die Behandlung mit einer individualisierten, zielgerichteten Flüssigkeits – und Kreislauftherapie ist grundsätzlich mit keinen Risiken verbunden, da keine zusätzlichen Interventionen oder unbekannte Medikamente bei Ihnen angewandt werden. Durch die Erhebung zusätzlicher Kreislaufparameter kann vielmehr eine genauere und auf Ihre Bedürfnisse spezifisch zugeschnittenere Therapie mit Flüssigkeiten und auch etwaigen Kreislaufmedikamenten erfolgen. Da es sich bei dem zusätzlich eingesetzten Gerät und den zusätzlich erhobenen Kreislaufparametern um etablierte, zugelassene und klinisch mittlerweile regelhaft eingesetzte Verfahren handeln, ist auch an dieser Stelle nicht von einem Risiko für Sie auszugehen. Auch ist nicht von einer Wechselwirkung mit anderen von Ihnen benötigten Medikamenten auszugehen.

6. Welche anderen Behandlungsmöglichkeiten gibt es außerhalb der Studie?

Zur Behandlung Ihrer Erkrankung stehen auch die folgenden Möglichkeiten zur Verfügung:
Die Alternative zu der individualisierten, zielgerichteten Flüssigkeits- und Kreislauftherapie ist die jeweilige Standardtherapie mit Flüssigkeiten und kreislaufwirksamen Medikamenten, die sich an Parametern wir z.B. Herzfrequenz und Blutdruck orientieren. Es sei an dieser Stelle nochmal darauf hingewiesen, dass die klassischen Parameter der Kreislaufüberwachung natürlich auch bei der individualisierten, zielgerichteten Flüssigkeits- und Kreislauftherapie berücksichtigt werden. Es werden hierbei zusätzlich jedoch weitere Parameter erhoben, die ebenfalls mit in die Therapieentscheidung einbezogen werden.

7. Wer darf an dieser klinischen Studie nicht teilnehmen?

An dieser klinischen Studie dürfen Sie nicht teilnehmen, wenn Sie gleichzeitig an anderen klinischen Studien oder anderen klinischen Forschungsprojekten teilnehmen.

8. Entstehen für mich Kosten durch die Teilnahme an der klinischen Studie? Erhalte ich eine Aufwandsentschädigung?

Durch Ihre Teilnahme an dieser klinischen Studie entstehen für Sie keine zusätzlichen Kosten.

9. Bin ich während der klinischen Prüfung versichert?

Da bei dieser Studie keine zusätzlichen Interventionen und lediglich etablierte Medikamente und Therapieverfahren eingesetzt werden, ist eine zusätzliche studienbedingte Patientenversicherung nicht erforderlich. Sie sind daher wie gewöhnlich auch über die Betriebshaftpflichtversicherung des Klinikums versichert.

10. Werden mir neue Erkenntnisse während der klinischen Studie mitgeteilt?

Sie werden über neue Erkenntnisse, die in Bezug auf diese klinische Studie bekannt werden und die für Ihre Bereitschaft zur weiteren Teilnahme wesentlich sein können, informiert. Auf dieser Basis können Sie dann Ihre Entscheidung zur weiteren Teilnahme an dieser klinischen Prüfung überdenken.

11. Wer entscheidet, ob ich aus der klinischen Studie ausscheide?

Sie können jederzeit, auch ohne Angabe von Gründen, Ihre Teilnahme beenden, ohne dass Ihnen dadurch irgendwelche Nachteile bei Ihrer medizinischen Behandlung entstehen.

Unter gewissen Umständen ist es aber auch möglich, dass der Prüfarzt oder der für die Studie Verantwortliche entscheidet, Ihre Teilnahme an der klinischen Prüfung vorzeitig zu beenden, ohne dass Sie auf die Entscheidung Einfluss haben. Die Gründe hierfür können z.B. sein:

- es wird die gesamte klinische Studie abgebrochen.
- nach Einschluss von 50% der Patienten stellt sich heraus, dass die individualisierte, zielgerichtete Flüssigkeits- und Kreislauftherapie keinerlei Vorteile bringt

12. Was geschieht mit meinen Daten?

Während der klinischen Studie werden medizinische Befunde und persönliche Informationen von Ihnen erhoben und in der Prüfstelle in Ihrer persönlichen Akte niedergeschrieben oder elektronisch gespeichert. Die für die klinische Studie wichtigen Daten werden zusätzlich in pseudonymisierter Form gespeichert, ausgewertet und gegebenenfalls weitergegeben. Pseudonymisiert bedeutet, dass keine Angaben von Namen

oder Initialen verwendet werden, sondern nur ein Nummern- und/oder Buchstabencode, evtl. mit Angabe des Geburtsjahres.

Die Daten sind gegen unbefugten Zugriff gesichert. Eine Entschlüsselung erfolgt nur unter den vom Gesetz vorgeschriebenen Voraussetzungen. Einzelheiten entnehmen Sie bitte der Einwilligungserklärung, die im Anschluss an diese Patienteninformation abgedruckt ist.

13. Was geschieht mit meinen Blutproben / Gewebeproben / Aufnahmen mit bildgebenden Verfahren (an die jeweilige Studie anpassen)?

Für die Studie selber werden keinerlei zusätzliche Blutproben, Gewebeproben oder Aufnahmen mit bildgebenden Verfahren durchgeführt.

14. An wen wende ich mich bei weiteren Fragen?

Beratungsgespräche an der Prüfstelle

Sie haben stets die Gelegenheit zu weiteren Beratungsgesprächen mit dem auf Seite 1 genannten oder einem anderen Prüfarzt.

Prüfstelle: Klinik für Anästhesiologie, Operative Intensivmedizin und Schmerztherapie der Justus-Liebig Universität Gießen
Prüfarzt: Univ. Prof. Dr. med. Michael Sander

„iPEGASUS –Individualisierte, zielgerichtete Herzkreislauf-Therapie bei großen bauchchirurgischen Operationen – Eine prospektive, randomisierte, klinische Studie“

Einwilligungserklärung

....
Name des Patienten in Druckbuchstaben

geb. am .... Teilnehmer-Nr. ....

Ich bin in einem persönlichen Gespräch durch den Prüfarzt

....
Name der Ärztin/des Arztes

ausführlich und verständlich über die zu prüfende Behandlungsmethode und die Vergleichsmethode sowie über Wesen, Bedeutung, Risiken und Tragweite der klinischen Studie aufgeklärt worden. Ich habe darüber hinaus den Text der Patienteninformation sowie die hier nachfolgend abgedruckte Datenschutzerklärung gelesen und verstanden. Ich hatte die Gelegenheit, mit dem Prüfarzt über die Durchführung der klinischen Prüfung zu sprechen. Alle meine Fragen wurden zufrieden stellend beantwortet.

Möglichkeit zur Dokumentation zusätzlicher Fragen seitens des Patienten oder sonstiger Aspekte des Aufklärungsgesprächs:

Ich hatte ausreichend Zeit, mich zu entscheiden.

Mir ist bekannt, dass ich jederzeit und ohne Angabe von Gründen meine Einwilligung zur Teilnahme an der Prüfung zurückziehen kann (mündlich oder schriftlich), ohne dass mir daraus Nachteile für meine medizinische Behandlung entstehen.

Datenschutz:

Mir ist bekannt, dass bei dieser klinischen Prüfung personenbezogene Daten, insbesondere medizinische Befunde über mich erhoben, gespeichert und ausgewertet werden sollen. Die Verwendung der Angaben über meine Gesundheit erfolgt nach gesetzlichen Bestimmungen und setzt vor der Teilnahme an der klinischen Prüfung folgende freiwillig abgegebene Einwilligungserklärung voraus, das heißt ohne die nachfolgende Einwilligung kann ich nicht an der klinischen Prüfung teilnehmen.

1. Ich erkläre mich damit einverstanden, dass im Rahmen dieser klinischen Studie personenbezogene Daten, insbesondere Angaben über meine Gesundheit, über mich erhoben und in Papierform sowie auf elektronischen Datenträgern bei/in .... (Institution/Ort der Aufzeichnung angeben) aufgezeichnet werden. Soweit erforderlich, dürfen die erhobenen Daten pseudonymisiert (verschlüsselt) weitergegeben werden an .... , den Verantwortlichen oder eine von diesem beauftragte Stelle zum Zwecke der wissenschaftlichen Auswertung.

2. Außerdem erkläre ich mich damit einverstanden, dass autorisierte und zur Verschwiegenheit verpflichtete Beauftragte des Verantwortlichen sowie die zuständigen Überwachungsbehörden in meine beim Prüfarzt vorhandenen personenbezogenen Daten, insbesondere meine Gesundheitsdaten, Einsicht nehmen, soweit dies für die Überprüfung der ordnungsgemäßen Durchführung der Studie notwendig ist. Für diese Maßnahme entbinde ich den Prüfarzt von der ärztlichen Schweigepflicht.

3. Ich bin bereits darüber aufgeklärt worden, dass ich jederzeit die Teilnahme an der klinischen Prüfung beenden kann. Im Fall eines solchen Widerrufs meiner Einwilligung, an der Studie teilzunehmen, erkläre ich mich damit einverstanden, dass die bis zu diesem Zeitpunkt gespeicherten Daten weiterhin verwendet werden dürfen, soweit dies erforderlich ist, um sicherzustellen, dass meine schutzwürdigen Interessen nicht beeinträchtigt werden. Falls ich meine Einwilligung, an der Studie teilzunehmen, widerrufe, müssen alle Stellen, die meine personenbezogenen Daten, insbesondere Gesundheitsdaten, gespeichert haben, unverzüglich prüfen, inwieweit die gespeicherten Daten zu dem vorgenannten Zweck noch erforderlich sind. Nicht mehr benötigte Daten sind unverzüglich zu löschen.

4. Ich erkläre mich damit einverstanden, dass meine Daten nach Beendigung oder Abbruch der Studie zehn Jahre aufbewahrt werden. Danach werden meine personenbezogenen Daten gelöscht, soweit nicht gesetzliche, satzungsmäßige oder vertragliche Aufbewahrungsfristen entgegenstehen (vertraglich vereinbarte Fristen müssen hier genannt werden).

6. Ich bin damit einverstanden, dass mein Hausarzt

   ....
   Name

   über meine Teilnahme an der klinischen Studie informiert wird (falls nicht gewünscht, bitte streichen).

Ich erkläre mich bereit,
an der oben genannten klinischen Studie
freiwillig teilzunehmen.

Ein Exemplar der Patienten-Information und -Einwilligung habe ich erhalten. Ein Exemplar verbleibt im Prüfzentrum.

....
Name des Patienten in Druckbuchstaben

....
Datum   Unterschrift des Patienten

Ich habe das Aufklärungsgespräch geführt und die Einwilligung des Patienten eingeholt.

....
Name des Prüfarztes/der Prüfärztin in Druckbuchstaben

....
Datum   Unterschrift des aufklärenden Prüfarztes/der Prüfärztin
