# Supplementary material for: Individualized, perioperative, hemodynamic goal-directed therapy in major abdominal surgery (iPEGASUS trial): study protocol for a randomized controlled trial
Source: Trials. 2018 May 9;19:273. doi: 10.1186/s13063-018-2620-9 (PMC5944092; doi:10.1186/s13063-018-2620-9)
Supplement: Supplementary file 4 — Table S1. Management of severe bleeding – Extract from the Guidelines from the European Society of Anaesthesiology. (PDF 258 kb) [file 13063_2018_2620_MOESM4_ESM.pdf]

<u>Table a1:</u> Overview of the Guidelines on Management of Severe Bleeding. Modified, based on the *Guidelines from the European Society of Anaesthesiology (ESA)* [21].

Optimising Macrocirculation<br>- aggressive and timely stabilisation of cardiac preload<br>- avoidance of hypervolemia with crystalloids or colloids […] beyond an optimal cardiac preload<br>- against the use of central venous pressure and pulmonary artery occlusion pressure as the only variables to guide fluid therapy, […] optimise preload during severe bleeding, dynamic assessment of fluid responsiveness and non-invasive measurement of cardiac output should be considered<br>- replacement of extracellular fluid losses with isotonic crystalloids in a timely and protocol-based manner<br>- haemodynamic stabilization with iso-oncotic colloids, such as human albumin and hydroxyethyl starch<br>- use of balanced solutions for crystalloids as a basic solute for iso-oncotic preparations
Transfusion triggers<br>- a target haemoglobin concentration of 7–9g/dl during active bleeding<br>- a restrictive transfusion strategy […] is beneficial in reducing exposure to allogeneic blood products
Oxygen fraction<br>- inspiratory oxygen fraction should be high enough to prevent arterial hypoxaemia in bleeding patients, while avoiding extensive hyperoxia (PaO2>26.7 kPa [200 mmHg])
Monitoring tissue perfusion<br>- repeated measurements of a combination of haematocrit/haemoglobin, serum lactate, and base deficit<br>- extended by measurement of cardiac output, dynamic parameters of volume status (e.g. stroke volume variation, pulse pressure variation) and central venous oxygen saturation
Coagulation management / Antifibrinolytics and tranexamic acid
Correction of confounding factors
