# Supplementary material for: Individualized, perioperative, hemodynamic goal-directed therapy in major abdominal surgery (iPEGASUS trial): study protocol for a randomized controlled trial
Source: Trials. 2018 May 9;19:273. doi: 10.1186/s13063-018-2620-9 (PMC5944092; doi:10.1186/s13063-018-2620-9)
Supplement: Supplementary file 5 — Table S2. Overview of Single Organ Outcome Failures and Severity Grading. (PDF 182 kb) [file 13063_2018_2620_MOESM5_ESM.pdf]

Table a2: Overview of the Single Organ Outcome Failures and their Severity Grading. Modified, based on the consensus statement of the ESA (European Society of Anesthesiology) -ESICM (European Society of Intensive Care Medicine) joint taskforce on perioperative outcome measures [22].

<table>
<tr><th>Single Organ Outcome Failure</th><th>Severity Grading</th></tr>
<tr><td>Anastomotic breakdown</td><td rowspan="17">Mild: Results in only temporary harm and would not usually require specific clinical treatment.

Moderate: More serious complication but one which does not usually result in permanent harm or functional limitation.
Usually requires clinical treatment.

Severe: Results in significant prolongation of hospital stay and/or permanent functional limitation or death. Almost always requires clinical treatment.</td></tr>
<tr><td>Arrhythmia</td></tr>
<tr><td>Cardiogenic pulmonary oedema</td></tr>
<tr><td>Deep vein thrombosis (DVT)</td></tr>
<tr><td>Gastrointestinal bleed</td></tr>
<tr><td>Infection, source uncertain</td></tr>
<tr><td>Laboratory confirmed bloodstream infection</td></tr>
<tr><td>Myocardial infarction</td></tr>
<tr><td>Pneumonia</td></tr>
<tr><td>Paralytic ileus</td></tr>
<tr><td>Postoperative haemorrhage</td></tr>
<tr><td>Pulmonary embolism (PE)</td></tr>
<tr><td>Stroke</td></tr>
<tr><td>Surgical site infection (superficial)</td></tr>
<tr><td>Surgical site infection (deep)</td></tr>
<tr><td>Surgical site infection (organ/space)</td></tr>
<tr><td>Urinary tract infection</td></tr>
<tr><td>Acute kidney injury (AKI)</td><td rowspan="3">Integrated in the definition.</td></tr>
<tr><td>Acute respiratory distress syndrome (ARDS)</td></tr>
<tr><td>Delirium</td></tr>
<tr><td>Cardiac arrest</td><td rowspan="2">Binary outcome (yes/no).</td></tr>
<tr><td>Myocardial injury after non-cardiac surgery (MINS)</td></tr>
</table>
