# Supplementary material for: Individualized, perioperative, hemodynamic goal-directed therapy in major abdominal surgery (iPEGASUS trial): study protocol for a randomized controlled trial
Source: Trials. 2018 May 9;19:273. doi: 10.1186/s13063-018-2620-9 (PMC5944092; doi:10.1186/s13063-018-2620-9)
Supplement: Supplementary file 1 — Ethical approval. (PDF 386 kb) [file 13063_2018_2620_MOESM1_ESM.pdf]

JUSTUS-LIEBIG UNIVERSITÄT GIESSEN

FACHBEREICH 11 MEDIZIN

Ethik-Kommission, Klinikstr. 29 (Alte Chirurgie), D-35385 Gießen

Prof. Dr. M. Sander
Klinik für Anästhesiologie, Intensivmedizin, Schmerztherapie
Klinikstr. 33
35385 Gießen

ETHIK-KOMMISSION
am Fachbereich Medizin
Vorsitz: Prof. H. Tillmanns

Klinikstr. 29 (Alte Chirurgie)
D-35385 Gießen
Tel.: (0641)99-42470 / 47660
ethik.kommission@pharma.med.uni-giessen.de

Gießen, 8. Februar 2017
Dr. Kr./

AZ.: **257/16**

Titel: *Individualised perioperative haemodynamic goal-directed therapy in major abdominal surgery (iPEGASUS-trial): a multi-centre, prospective, randomised trial.*

Meeting19.01.2017

Dear Prof. Sander,

The referred project was discussed in detail during the January meeting of the ethics committee (presentation: MD Miss. Dagmar Schulte). The project is a prospective evaluation of a goal directed hemodynamic therapy in comparison to the standard therapy in the perioperative management of major elective visceral-surgical procedures (estimated surgery time >120 min.).
The design of the study aims to randomize two study groups, involving the minimal-invasive monitoring system ProAQT® in the extended setting (Pulsion Medical SE, Munich, Germany) with a surveillance- and therapy algorithm planned by the proposers (the device will be disposed, CE-certificate is present).
No additional strains or risks will result for the patients. They will be informed about the study and will be asked for consent.

No major scientific criticism was raised in the discussion, however the biometric expert encouraged that potential sources of interference (e.g. type and length of antibiotic treatment) could be minimized by exact stratification of the included patients to generate more homogeneous study groups.
Formal points of criticism have been not addressed.

The ethics committee asks for reconsideration of the biometrics, but raises no objection against performing the project.

With best regards

Prof. Dr. H. Tillmanns
Chair of the ethics committee
